# 24-month outcomes of a combined 180-degree phacogoniotomy with KDB procedure

**DOI:** 10.3389/fopht.2026.1910636

**Published:** 2026-08-19

**Authors:** Panagiotis Tsoutsanis, Ananth Ranjit, Afifa Islam, Siddharth Agrawal

**Affiliations:** 1 Department of Ophthalmology, Northern Care Alliance NHS Foundation Trust, Rochdale Infirmary, Rochdale, United Kingdom; 2 School of Medical Sciences, Faculty of Biology, Medicine and Health, The University of Manchester, Manchester, United Kingdom

**Keywords:** cataract surgery, glaucoma, KDB goniotomy, MIGS, phacogoniotomy

## Abstract

**Purpose:**

We aimed to evaluate the 24-month efficacy and safety of combined 180-degree phacogoniotomy with Kahook Dual Blade (KDB) in patients with various forms of glaucoma.

**Methods:**

This retrospective, single-centre study included 66 eyes from 66 patients who underwent combined phacogoniotomy with KDB surgery at Rochdale Infirmary, UK, between November 2021 and January 2023. Patients with moderate-to-advanced open-angle, secondary, or narrow-angle glaucoma were included. Primary outcomes were changes in mean intraocular pressure (IOP) and number of IOP-lowering medications from baseline to 24 months. Secondary outcomes included surgical success, defined according to the criteria of Dorairaj et al. and the American Academy of Ophthalmology (AAO) criteria.

**Results:**

Mean IOP significantly decreased from 19.4 ± 6.6 mmHg at baseline to 14.1 ± 4.3 mmHg at 24 months, representing a mean reduction of 5.3 mmHg (27%) (p < 0.0001). The mean number of IOP-lowering medications was reduced from 3.0 ± 0.7 to 1.7 ± 1.2 at 24 months, a mean reduction of 1.3 medications (43%) (p < 0.0001). At 24 months, 92% of eyes achieved success by Dorairaj’s criteria, and 76% achieved qualified success and 19% absolute success according to AAO standards. Visual acuity improved slightly but not significantly.

**Conclusion:**

Combined 180-degree phacogoniotomy with KDB provides a significant and sustained 24-month reduction in intraocular pressure and medication burden, with a favourable safety profile. These findings support phacogoniotomy with KDB as an effective minimally invasive option for patients with coexisting cataract and glaucoma.

## Introduction

Glaucoma is a heterogeneous chronic progressive optic neuropathy, one of the leading causes of blindness around the world. It is thought to be a multifactorial disease, in which the intraocular pressure (IOP) is identified as a modifiable risk factor. Research has shown that lowering IOP can slow down or stop disease progression (1–3). The conventional approach to lowering IOP involves commencing with the safer and least invasive approaches such a topical medication (drops) or selective laser trabeculoplasty (4) and progressively moving to more invasive management, which includes surgical interventions (5) (6).

Surgical procedures aiming to lower IOP are by no means new to the field. At least since the 1850s, surgical innovators such as Von Graefe and De Wecker have described early iridectomy and sclerotomy procedures, respectively. Later in the 19th century, modifications to these procedures were proposed, and it was in the 20th century that the field of glaucoma surgical innovations truly flourished. The early versions of virtually all glaucoma procedures used today, including surgeries involving shunts, fistula-forming operations, and cyclodialysis, were first proposed and demonstrated in the 20th century, with some of them, such as trabeculectomy, being used almost unchanged to this day (7).

Since the beginning of the 21st century, building on knowledge created in the century prior, there has been a new wave of glaucoma procedures called minimally invasive glaucoma surgery (MIGS). The principle behind MIGS is to offer an IOP-lowering procedure that, although it shows a more moderate IOP reduction when compared with traditional glaucoma surgeries (such as trabeculectomy), has a more favourable safety profile with lower risk of complications. A variety of MIGS have been invented and are often categorised according to the anatomical structure they are targeting.

MIGS targeting the Schlemm’s canal can work by offering a trabecular bypass using a stent, dilating the Schlemm’s canal, or creating a trabeculotomy. MIGS procedures can also shunt aqueous into the suprachoroidal space or into the subconjunctival space. Finally, others can reduce aqueous production by targeting the ciliary body (8).

Our study focuses on the Kahook Dual Blade (KDB) goniotomy procedure paired with phacoemulsification and intraocular lens (IOL) surgery (phacogoniotomy).

The KDB goniotomy is a Schlemm’s canal-based procedure designed to remove trabecular meshwork (TM) tissue and restore the natural outflow of aqueous humour. Unlike a traditional goniotomy knife, which simply incises the TM and leaves residual flaps, the KDB excises a complete strip of TM, thereby creating a direct pathway for aqueous humour to flow from the anterior chamber into the Schlemm’s canal. This approach avoids the need for bleb formation or permanent implants. The KDB is a handheld knife specifically engineered for precise TM excision. Its design includes a pointed distal tip that pierces the TM and enters Schlemm’s canal, a 230-µm-wide footplate that stabilises the instrument within the canal, and a ramp leading to two elevated parallel blades. As the device advances along the Schlemm’s canal, the TM is engaged, stretched, and guided up the ramp, where the dual blades cleanly remove a narrow strip of tissue. The diameter of the handle allows the surgeon to effectively treat the angle through a single clear corneal incision by pivoting the shaft within it (9, 10).

In our study, we present the 24-month results in terms of IOP reduction and IOP-lowering medication alterations in 66 patients who underwent 180-degree phacogoniotomy with KDB.

## Methods

This was a retrospective, single-centre study of patients who underwent combined phacogoniotomy with KDB at Rochdale Infirmary, Northern Care Alliance NHS Foundation Trust, UK. Surgical procedures were performed between November 2021 and January 2023, with a planned follow-up of 24 months. The study adhered to the ethical standards of the institution and complied with the tenets of the Declaration of Helsinki.

Eligible patients were aged 18 years or older with a diagnosis of moderate-to-advanced open-angle glaucoma, ocular hypertension (OHT), secondary glaucoma (pigmentary, pseudoexfoliative, steroid-induced, or uveitic), and narrow-/closed-angle glaucoma. Moderate and advanced glaucoma were defined according to the Hodapp–Parrish–Anderson classification using visual field mean deviation (MD), with moderate glaucoma defined as an MD between −6 and −12 dB and advanced glaucoma as an MD worse than −12 dB. Exclusion criteria comprised any prior glaucoma surgery, prior ruthenium plaque brachytherapy, ocular trauma, or corneal pathology that could interfere with accurate IOP measurement.

Clinical data were obtained through retrospective review of the OpenEyes electronic medical record system. Parameters collected included demographics, ophthalmic history, best-corrected visual acuity (BCVA) using the LogMAR scale, intraocular pressure (IOP) measured by Goldmann applanation tonometry, number of IOP-lowering medications, and postoperative complications. IOP and medication data were recorded at baseline, 1 month, 3 months, 6 months, 12 months, and 24 months postoperatively.

All surgeries were performed by a single consultant glaucoma specialist (S.A.) under sub-Tenon anaesthesia. Before the procedure, the patients had been counselled properly that unlike simple phacoemulsification with IOL, their vision would remain blurred for a few weeks secondary to the hyphaema and intracameral triamcinolone usage.

Standard phacoemulsification with posterior chamber intraocular lens implantation was performed first. Subsequently, KDB goniotomy was performed under direct gonioscopic visualisation using a surgical goniolens. The Kahook Dual Blade (New World Medical, Rancho Cucamonga, CA, USA) was introduced through the same corneal incision as the phacoemulsification and advanced along Schlemm’s canal to excise 180° of trabecular meshwork (superonasal, inferonasal, or inferotemporal quadrants). At the end of the procedure, intracameral cefuroxime was administered, followed by intracameral and sub-Tenon triamcinolone.

The patients with narrow angles were treated with goniosynechialysis using healon greater viscosity (HGV) viscoelastic and/or an iris repositor prior to performing the goniotomy. One eye underwent 60 degrees whilst the other 45 degrees of goniosynechialysis, both over the nasal quadrant.

Postoperative care consisted of intensive topical corticosteroid therapy for 2 months. Patients with ocular surface disease received preservative-free dexamethasone 0.1% 10 times daily, whilst those without ocular surface disease received prednisolone acetate 1% (Pred Forte) 8 times daily for 2 months. Preservative-free chloramphenicol eye drops were prescribed 4 times daily for 4 weeks, atropine 1% once daily for 2 weeks, and chloramphenicol ointment nightly for 2 weeks. Oral acetazolamide was prescribed for 2–3 days postoperatively, subject to renal function. Preoperative glaucoma medications were continued until the first postoperative review, which occurred within 1 week of surgery. Thereafter, glaucoma medications were withdrawn sequentially at the discretion of the lead surgeon, typically by discontinuing one glaucoma medication each month. Patients were instructed to stop one glaucoma medication 1 week before each scheduled follow-up visit to allow assessment of the intraocular pressure response following medication withdrawal.

The primary outcomes were changes in mean IOP and medication from baseline. Secondary outcomes included surgical success at 12 and 24 months as defined by Dorairaj et al. (9) and the American Academy of Ophthalmology’s (AAO’s) Glaucoma Preferred Practice Pattern (PPP) Committee recommendations (11). The Dorairaj criteria stipulate ≥20% IOP reduction from baseline, or a reduction of ≥1 IOP-lowering medication from baseline. AAO PPP criteria were defined as either a reduction of at least one glaucoma medication from baseline without any rise in intraocular pressure (IOP), or an IOP ≤21 mmHg with a ≥20% decrease from baseline and no increase in glaucoma therapy. Additional glaucoma surgery, laser intervention, hypotony, or loss of light perception were considered treatment failures. Furthermore, a final IOP of ≤18 mmHg was required to meet the success criteria. Qualified success was defined as fulfilling these conditions with or without the use of glaucoma medication, whereas absolute success required meeting the same criteria without any IOP-lowering treatment.

As this was a retrospective, real-world analysis, no formal sample size calculation was performed. Statistical analysis was conducted using GraphPad Prism (GraphPad Software, San Diego, CA, USA). Data normality was assessed using histograms and the Shapiro–Wilk test (W > 0.05 indicating normal distribution). Paired t-tests were used to compare mean changes from baseline at each follow-up time point, with Bonferroni correction applied to adjust for multiple comparisons. Linear mixed-effects models using restricted maximum likelihood (REML) estimation were applied to analyse longitudinal trends in intraocular pressure (IOP) and number of medications across multiple time points. Time was modelled as a fixed effect and subject as a random effect, accounting for within-subject correlation and allowing for missing data due to missed follow-up. A p-value < 0.05 was considered statistically significant.

## Results

A total of 66 eyes from 66 patients were included in the analysis, with a mean age of 74 ± 6 years. The majority of patients were diagnosed with primary open-angle glaucoma (POAG) (92%), and the mean baseline best-corrected visual acuity (BCVA) was 0.28 ± 0.36 LogMAR. Baseline characteristics are featured in **Table 1**.

**Table 1:** Baseline characteristics.

Characteristics	N = 66
Age (years)	74 ± 6
Sex M:F	30:36
Diagnosis POAG: Secondary: OHT: NTG: Narrow Angle	61:1:1:1:2
POAG (N = 61) Moderate: Advanced	33:28
BCVA (LogMAR ± SD)	0.28 ± 0.36
Mean follow-up duration (months)	25.5 ± 5.6

**Figure 1:**
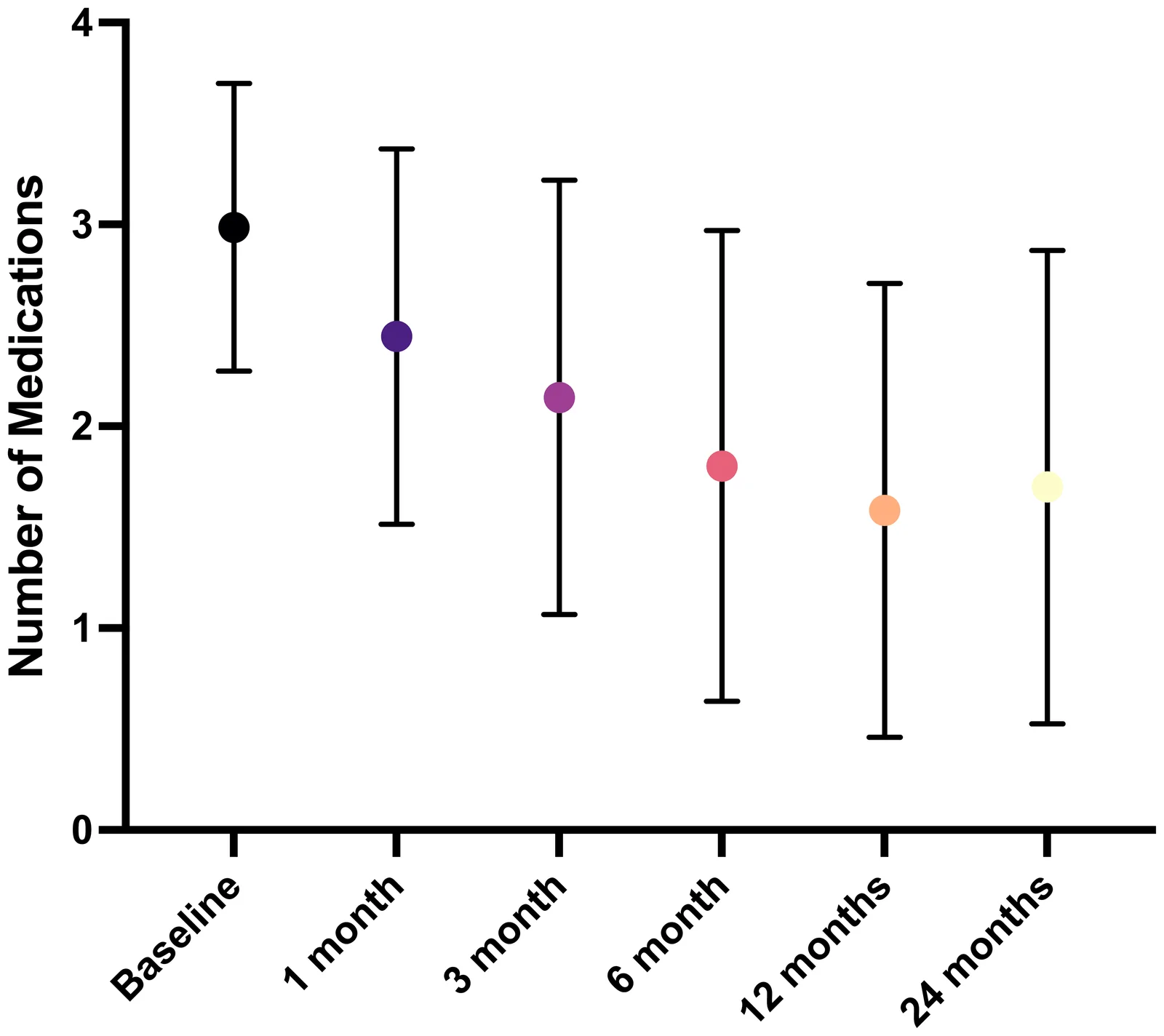
Mean intraocular pressure at baseline and post-goniotomy at different time points.

The mean IOP decreased significantly from 19.4 ± 6.6 mmHg at baseline to 12.5 ± 3.2 mmHg at 12 months and 14.1 ± 4.3 mmHg at 24 months (**Table 2**). A mixed-effects model (REML) with time as a fixed effect and subject as a random effect demonstrated a significant change in mean IOP over time [F(2.879, 175.6) = 34.45, p < 0.0001, Geisser–Greenhouse ϵ = 0.576]. Pairwise comparisons using Bonferroni-adjusted t-tests confirmed significant reductions in IOP at all postoperative time points compared with baseline (p < 0.0001 for all; **Table 3**). **Figure 1** demonstrates a graphical representation of mean IOP after KDB goniotomy.

**Table 2:** Intraocular pressure and IOP-lowering medication pre- and post-interventions.

Timepoint	Baseline (n = 66)	1 month (n = 63)	3 months (n = 63)	6 months (n = 61)	12 months (n = 61)	24 months (n = 63)
IOP (Mean ± SD)	19.4 ± 6.6	13.2 ± 4.5	12.6 ± 3.5	12.6 ± 3.7	12.5 ± 3.2	14.1 ± 4.3
No. of drops (Mean ± SD)	3.0 ± 0.7	2.4 ± 0.9	2.1 ± 1.1	1.8 ± 1.2	1.6 ± 1.1	1.7 ± 1.2

**Table 3:** Baseline comparison of IOP.

Comparison	Mean IOP difference (mmHg)	Adjusted p-value (Bonferroni) p Value	95% CI
Baseline – 1 month	-5.9	<0.0001	-7.8 - -4.0
Baseline – 3 months	-6.7	<0.0001	-8.3 - 5.0
Baseline – 6 months	-6.8	<0.0001	-8.4 - -5.1
Baseline – 12 months	-6.9	<0.0001	-8.7 - -5.2
Baseline – 24 months	-5.4	<0.0001	-7.2 - -3.6

**Figure 2:**
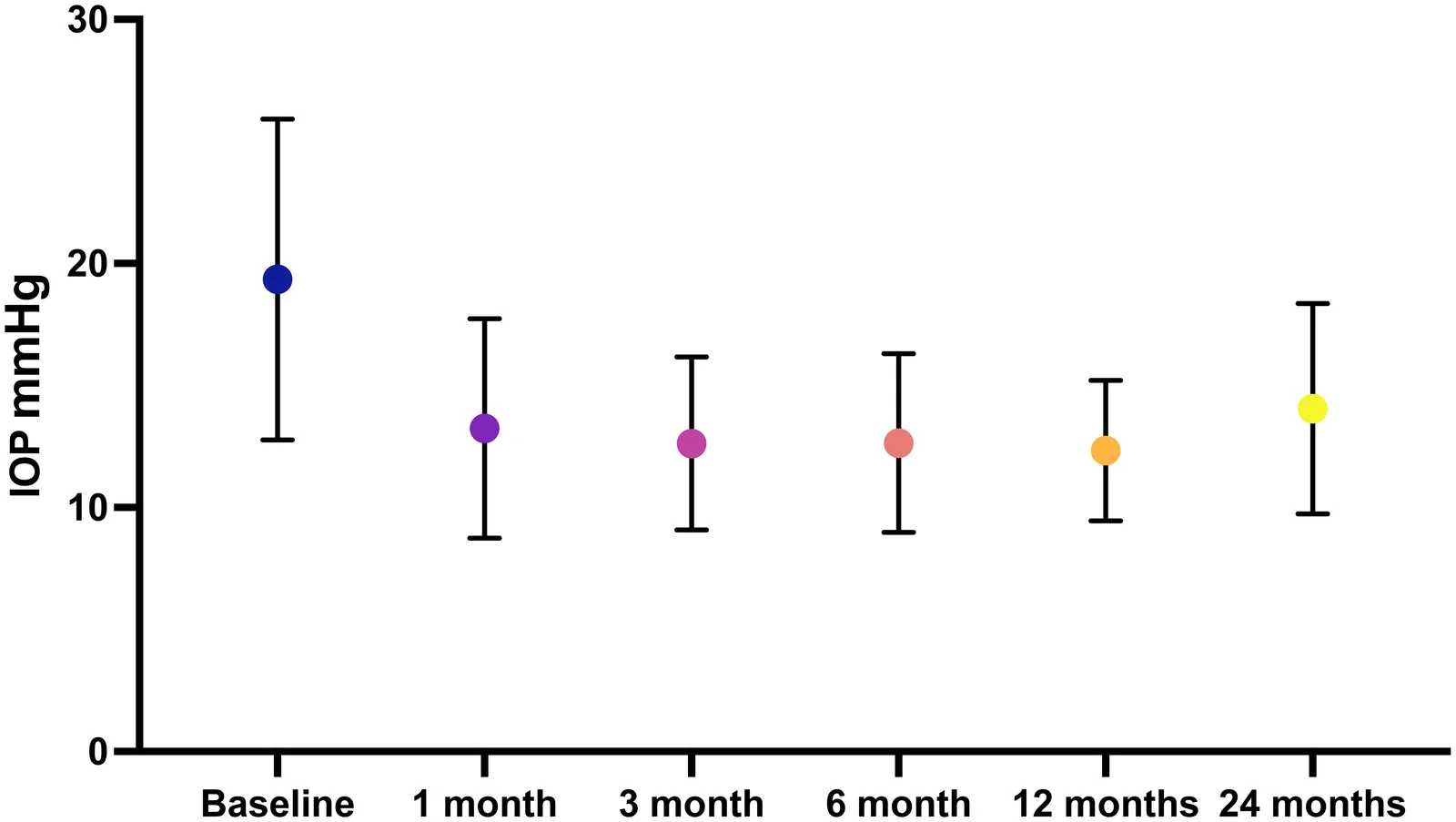
Mean number of medications used at baseline and post-goniotomy at different time points.

The mean number of IOP-lowering medications decreased from 3.0 ± 0.7 at baseline to 1.6 ± 1.1 at 12 months and 1.7 ± 1.2 at 24 months (**Table 4**). Mixed-effects modelling demonstrated a statistically significant reduction in the number of topical medications over time (F(2.881, 175.5) = 144.4, p < 0.0001, Geisser–Greenhouse ϵ = 0.576). Changes in IOP-lowering medications are represented in **Figure 2**.

**Table 4:** Moderate glaucoma - intraocular pressure and IOP-lowering medication pre- and post-interventions.

Timepoint	Baseline (n = 33)	1 month (n = 32)	3 months (n = 33)	6 months (N = 32)	12 months (N = 30)	24 months (N = 31)
IOP (Mean ± SD)	19.1 ± 5.4	13.6 ± 4.9	12.9 ± 3.4	12.3 ± 3.5	13.2 ± 3.4	14.4 ± 3.9
No. of drops (Mean ± SD)	3.1 ± 0.6	2.4 ± 1.0	2.1 ± 1.1	1.8 ± 1.1	1.5 ± 1.1	1.7 ± 1.1

The mean BCVA improved from 0.28 ± 0.36 LogMAR at baseline to 0.20 ± 0.44 LogMAR at 24 months; however, that improvement was not statistically significant (p = 0.06, 95% CI = –0.15 to 0.002). We hypothesise that the improvement in BCVA was relatively small after cataract surgery due to the baseline BCVA being relatively good (0.28 ± 0.36 LogMAR) preoperatively in our study cohort. Many of our patients, however, did note that their subjective visual perception had improved after surgery (brightness, colour vision).

Postoperatively, most of the patients had hyphaema at 1 week after surgery, which resolved at 2–4 weeks after surgery. The rest of the complications were infrequent with only one patient requiring anterior chamber washout for hyphaema, and a single IOP spike was recorded (IOP >30 mmHg in the first follow-up). No cases of cyclodialysis cleft or hypotony were recorded. Two patients underwent trabeculectomy for IOP above 18 mm Hg, with or without evidence of glaucoma progression on visual field or optical coherence tomography (OCT) over the 24-month follow-up period.

### Sub-level analysis

#### Moderate glaucoma (N = 33)

The mean IOP decreased significantly from 19.1 ± 5.4 mmHg at baseline to 13.2 ± 3.4 mmHg at 12 months and 14.4 ± 3.9 mmHg at 24 months (**Table 4**). A mixed-effects model (REML) with time as a fixed effect and subject as a random effect demonstrated a significant change in mean IOP over time [F(3.02, 92.36) = 18.33, p < 0.0001, Geisser–Greenhouse ϵ = 0.604].

The mean number of IOP-lowering medications decreased significantly from 3.1 ± 0.6 at baseline to 1.5 ± 1.1 at 12 months and 1.7 ± 1.1 at 24 months (**Table 4**). Time had a significant effect on medication use [F(3.02, 93.03) = 21.40, p < 0.0001, Geisser–Greenhouse ϵ = 0.604].

#### Advanced glaucoma (N = 28)

The mean IOP decreased significantly from 18.9 ± 4.9 mmHg at baseline to 11.8 ± 2.9 mmHg at 12 months and 13.4 ± 4.3 mmHg at 24 months (**Table 5**). A mixed-effects model (REML) with time as a fixed effect and subject as a random effect demonstrated a significant change in mean IOP over time [F(3.20, 78.72) = 14.33, p < 0.0001, Geisser–Greenhouse ϵ = 0.640].

**Table 5:** Advanced glaucoma - intraocular pressure and IOP-lowering medication pre- and post-interventions.

Timepoint	Baseline (n = 28)	1 month (n = 27)	3 months (n = 25)	6 months (N = 24)	12 months (N = 25)	24 months (N = 27)
IOP (Mean ± SD)	18.9 ± 4.9	13.3 ± 4.1	12.1 ± 4.0	12.8 ± 3.8	11.8 ± 2.9	13.4 ± 4.3
No. of drops (Mean ± SD)	2.9 ± 0.8	2.4 ± 1.0	2.3 ± 1.1	1.9 ± 1.3	1.8 ± 1.2	1.9 ± 1.2

The mean number of IOP-lowering medications decreased significantly from 2.9 ± 0.8 at baseline to 1.8 ± 1.2 at 12 months and 1.9 ± 1.2 at 24 months (**Table 5**). There was a significant effect of time on medication use [F(2.26, 55.67) = 7.59, p = 0.0008, Geisser–Greenhouse ϵ = 0.453].

In patients with baseline IOP <15 mmHg (n = 16), mean IOP showed no statistically significant change over time across 24 months [F(2.305, 32.72) = 0.87, p = 0.44]. The mean IOP was 13.1 ± 1.5 mmHg at baseline and 13.2 ± 3.5 mmHg at 24 months. In contrast, there was a significant overall reduction in the number of topical medications over time [F(1.846, 26.58) = 11.68, p = 0.0003], decreasing from 3.0 ± 0.6 at baseline to 1.0 ± 1.0 at 24 months.

In patients with baseline IOP 15–20 mmHg (n = 28), mean IOP decreased significantly over time [F(3.65, 91.95) = 26.87, p < 0.0001], falling from 18.0 ± 1.5 mmHg at baseline to 14.1 ± 4.4 mmHg at 24 months. The number of topical medications also reduced significantly across follow-up [F(2.85, 71.8) = 12.6, p < 0.0001], from 3.0 ± 0.6 at baseline to 1.8 ± 1.2 at 24 months.

In patients with baseline IOP between 21 and 25 mmHg (n = 15), mixed-effects modelling showed a significant reduction in mean IOP over time [F(3.20, 41.53) = 15.05, p < 0.0001], with mean values decreasing from 21.9 ± 0.9 mmHg at baseline to 14.4 ± 4.6 mmHg at 24 months. A significant reduction in the number of topical medications was also observed across follow-up [F(2.66, 34.6) = 6.02, p = 0.0028], from 2.8 ± 0.9 to 1.9 ± 1.0 at 24 months.

A small number of patients had a baseline IOP between 26 and 30 mmHg (n = 3). Mean IOP decreased markedly over time [F(1.31, 2.62) = 125.7, p = 0.0026], from 28.0 ± 1.0 mmHg at baseline to 15.7 ± 3.1 mmHg at 24 months. However, there was no statistically significant change in the number of topical medications used over time [F(1.214, 2.428) = 1.514, p = 0.33], which decreased from 3.3 ± 0.6 to 2.3 ± 1.2.

In eyes with baseline IOP > 30 mmHg (n = 4), mixed-effects modelling demonstrated a significant treatment effect on IOP reduction over time [F(1.513, 4.236) = 13.61, p = 0.0160], with mean IOP decreasing from 38.0 ± 1.0 mmHg at baseline to 14.5 ± 7.3 mmHg at 24 months. Although a reduction in the number of topical medications was observed (from 3.5 ± 0.6 to 2.0 ± 1.4), this change did not reach statistical significance [F(1.794, 5.024) = 5.82, p = 0.051].

#### Success outcomes at 12 months

By Dorairaj’s criteria, 90.2% of eyes achieved success (≥20% IOP reduction or ≥1 medication fewer than baseline). By AAO PPP criteria, 85% achieved qualified success, and 23% achieved absolute success (IOP ≤ 18 mmHg without medication).

#### Success outcomes at 24 months

At 24 months, 92% achieved qualified success by Dorairaj’s definition, whilst according to AAO PPP criteria, 76% achieved qualified success and 19% achieved absolute success.

## Discussion

Minimally invasive glaucoma surgery (MIGS) refers to a group of procedures which aim to reduce IOP and medication burden for patients whilst offering a safer risk profile and faster recovery times than traditional glaucoma procedures (12, 13).

Our study investigated the change in IOP together with medication use for a cohort of 66 patients comprised of 61 POAG, 1 secondary open-angle glaucoma, 1 ocular hypertension (OHT), 1 normal tension glaucoma (NTG); and 2 narrow-angle glaucoma treated with 180-degree phacogoniotomy with KDB. All surgeries were performed in the eye unit of Rochdale Infirmary and patients were followed at eight different time points, namely, at 1 week and 1, 2, 3, 6, 12, 18, and 24 months after surgery.

The Kahook Dual Blade (KDB) procedure excises a strip of the trabecular meshwork (TM) across several clock hours, enhancing aqueous outflow into the Schlemm’s canal. Since approximately 75% of aqueous humour drains through the TM and Schlemm’s canal, this area represents an appealing target for minimally invasive glaucoma surgery. Unlike other goniotomy-based techniques such as the trabectome (Neomedix, Tustin, CA, USA), the gonioscopy-assisted transluminal trabeculotomy (GATT), and traditional trabeculotomy, which only incise the TM, the KDB method may also remove the dissected tissue, and this additional step may theoretically lower the risk of scarring and reduce the likelihood of surgical failure (10, 14, 15).

Over the years, there have been various studies comparing different MIGS procedures with one another, comparing their combination with cataract surgery or their use as a stand-alone procedure. Our study’s results compare favourably with other studies that measured postoperative intraocular pressure (IOP) and medication reduction after procedures involving cataract surgery paired with MIGS.

In a study by Dorairaj et al. involving 58 patients (open-angle glaucoma, normal-tension glaucoma, exfoliation glaucoma, and pigmentary glaucoma) undergoing phacogoniotomy with KDB, the preoperative mean IOP (± SD) was 16.8 mmHg (± 0.6), and they used an average of 1.6 (± 0.2) drops. At the 12-month follow-up after surgery, the IOP showed a mean reduction of 4.4 mmHg (± 0.3), and the drops were reduced by 0.8 (± 0.1). However, there was no mention of the number of degrees to which KDB goniotomy was performed.

An interesting retrospective case-control study by Magacho et al. (16) compared the outcomes in terms of postoperative IOP and medication use between two open-angle glaucoma patient cohorts (each comprised of 36 eyes), with a mean age of 68.7 years. One cohort underwent standard combined phacogoniotomy with KDB surgery (GI; 30 degrees treated) whilst the other underwent extended phacogoniotomy with KDB surgery (GII; 150 degrees treated). The study followed the patients for 12 months, and although it did not have the 24-month results that would be comparable to our study, it is interesting to see the difference based on the degrees treated with KDB.

Preoperatively, IOP was comparable between the two groups with GI IOP (± SD) 17.0 mmHg (± 2.7) and GII 17.5 mmHg (± 4.2) (p = 0.5). At 12 months post-surgery, there was a significant reduction for both groups with the GI IOP being 13.5 mmHg (± 2.8), a reduction of 3.5 mmHg, and GII IOP being 12.3 mmHg (± 2.7), thus a reduction of 5.2 mmHg (p = 0.06). The mean number of glaucoma medications (± SD) decreased in both groups: from 1.7 (± 0.9) to 0.4 (± 0.8) in GI (p < 0.001), being a 1.3 absolute reduction, and from 2.1 (± 0.8) to 0.4 (± 0.7) in GII (p < 0.001), making it a 1.7 drop reduction.

In a study by Gedde et al., success was defined based on AAO PPP Committee recommendations (11), with qualified success achieved in 77.1% of the eyes in GI and in 94.4% in GII (p = 0.03), whereas absolute success was achieved in 68.6% of the eyes in GI and in 69.4% in GII (p = 0.7).

In our study, the mean pre-intervention IOP (± SD) was 19.4 mmHg (± 6.6), and the mean number of drops used pre-intervention was 3 (± 0.7), meaning that both the starting IOP and the number of drops used were significantly higher than those reported in the studies by Dorairaj et al. and Magacho et al.

At the 12-month point of our study, the mean IOP was reduced by 6.9 mmHg (95% CI: -8.7 to -5.1; P <0.0001) and the drops by 1.4 (SD ±1.1), meaning that these reductions, although higher absolute terms, are comparable to the other two studies.

Using the same recommendations about success, as used in the Magacho et al. study, at 12 months, our cohort achieved a lower qualified success at 85% compared with GII and lower absolute success at 23% compared with both their GI and GII groups. However, due to the nature of our cohort with the mean age being higher (74 ± 6), starting with a higher IOP and more medications at baseline, it is by design that the success percentage would be lower as this metric is very much dependent on final IOP being lower than 18 and patients being medication-free. Furthermore, our patient cohort had moderate-to-advanced glaucoma, unlike the study by Magacho et al., where the patients had early or moderate glaucoma. Multiple studies have shown that chronic use of glaucoma-lowering medications with preservatives such as benzalkonium chloride has detrimental long-term effects on the TM, causing cell apoptosis and scarring (17–20). Our patient cohort falls into that category, and we can assume that the health of the TM leads to less reduction in IOP or medication burden when compared with eyes with less severe glaucoma that perhaps have not had the same history of glaucoma and medication use.

Further comparative data come from two large multicentre studies by Song et al. and Zhang et al., who investigated the effect of goniotomy length on surgical outcomes in POAG, both with and without combined phacoemulsification (21, 22). In the study by Song et al., 44 eyes underwent combined 120-degree phacogoniotomy (baseline IOP 26.8 ± 8.4 mmHg), achieving a mean IOP reduction of 12.8 ± 9.9 mmHg (39.4%) at a mean follow-up of 7.2 months, whilst their 360-degree combined cohort (n = 30, baseline IOP 28.8 ± 6.0 mmHg) achieved a reduction of 13.8 ± 7.2 mmHg (46.0%) at a mean follow-up of 10.0 months. In their subsequent larger study, Zhang et al. extended this comparison to include a 240-degree arm, reporting IOP reductions of 9.4 ± 9.3 mmHg, 12.0 ± 9.9 mmHg, and 11.9 ± 9.6 mmHg in the combined 120-, 240-, and 360-degree groups, respectively, with no significant difference in efficacy found between goniotomy lengths. Both studies found comparable reductions in medication burden across all goniotomy lengths, with a median of zero medications required at final follow-up.

Although our study analysed IOP and medication reduction at different intervals and a different patient cohort, focusing exclusively on moderate-to-advanced glaucoma rather than only on POAG, some useful comparisons can nevertheless be made with certain arms of these studies.

The absolute IOP reduction in our cohort was smaller than that reported in these two studies, and this is best explained by our lower baseline IOP (19.4 ± 6.6 mmHg) compared with their combined-surgery cohorts (25.1–28.8 mmHg); our percentage IOP reductions of 36% at 12 months and 27% at 24 months are comparable with the percentage reductions reported by Song et al. and Zhang et al., whilst our final IOP values were lower in absolute terms than those achieved in either study. Notably, however, our medication reduction was less pronounced, with a mean of 1.7 ± 1.2 drops remaining at 24 months compared with a median of zero medications in the comparator studies’ phacogoniotomy arms. Importantly, both studies found no significant difference in IOP-lowering efficacy between 120-, 240-, and 360-degree goniotomy lengths, a finding that is consistent with the comparison drawn earlier with Magacho et al. (16) and that gives us further support to the notion that the extent of trabecular meshwork treated may not be the principal determinant of surgical success.

Other than the KDB goniotomy, other commonly used MIGS are the iStent, iStent inject, and Hydrus microstent.

A comparative study by Holmes et al. (23) analysed 334 eyes (mild to moderate open-angle glaucoma, normal-tension glaucoma, and ocular hypertension) undergoing phacoemulsification paired with either Hydrus microstent (120 eyes) or iStent inject (224 eyes) implantation. The preoperative mean IOP (± SD) was 18.1 mmHg (± 5.5) for the Hydrus and 16.3 mmHg (± 4.4) for the iStent inject, and the mean number of glaucoma drops (± SD) used was 2.1 (± 1.2) and 1.5 (± 1.2), respectively. At the 24-month follow-up, the Hydrus cohort had a mean reduction in IOP (95% CI) of 3 mmHg (-4, -2) and reduction in drops (95% CI) by 0.8 (-1.1, -0.6). At the same time point, the iStent inject group had a mean IOP reduction of 2.2 (-2.8, -1.6) and the same medication reduction of 0.8 (-0.9, -0.6).

This study following the patients 2 years after surgery is comparable to ours, where, at the 24-month point, we measured a mean reduction in IOP of 5.4 mmHg (95% CI: -7.2 to -3.6; P <0.0001).

Finally, a study by Barkander et al. (14) compared the 24-month efficacy of 153 eyes (cohort made up of POAG, pseudoexfoliation, and pigmentary glaucoma cases) undergoing either iStent implantation or KDB goniotomy paired with phacoemulsification and IOL. Although the authors do not mention the exact number of degrees treated with the KDB goniotomy, they note that the KDB knife removed one-third to one-fourth of TM nasally, and therefore we can safely assume that fewer degrees than the 180-degrees of our cases were treated. Most likely their extent is more comparable to the GI group from the Magacho et al. study (which was 30 degrees). The 56 eyes that received the iStent implant had a baseline mean IOP (± SD) of 20.3 mmHg (± 6.1) and a mean number of drops (± SD) used of 3 (± 0.9), whilst the 97 eyes forming the KDB goniotomy cohort had a comparable baseline IOP of 20.1 (± 6.1) and the number of drops used was 2.3 (± 1.0). At 24 months postoperatively, the iStent cohort had an IOP reduction of 6.1 mmHg (± 4.1) and a drop reduction of 0.4 (± 1.1), whilst the KDB group had IOP lowering by 5.4 mmHg (± 3.6), and the number of drops used was reduced by 0.8 (± 1.3).

It is therefore interesting to see that our study’s IOP reduction at 24 months was identical to that of their KDB group, but our study had a much smaller medication burden, as in our study, the drop use decreased from 3 (± 0.7) drops down to 1.7 (± 1.2) drops. This difference could be explained by the patient characteristics, inter-clinician variability in medication choice, or due to the larger degree of TM treated in our cases.

One of the risk factors of goniotomy failure is the scarring of the area where the TM is excised, which can still happen despite the special design of the KDB knife. It would be interesting to see other studies comparing small-extent KDB goniotomy to larger-extent goniotomy over multiple years to assess whether a higher extent of treatment can provide more sustained long-term results.

Despite the excellent results that can be achieved with MIGS, some glaucoma patients may require incisional surgery with trabeculectomy or aqueous shunt insertion.

A cross-sectional multicentre retrospective study by Kirwan et al. (24) included 428 eyes of 395 patients with open-angle glaucoma without any previous incisional glaucoma surgery that underwent trabeculectomy. Their results showed that the IOP (mean ± standard deviation) decreased from 23 ± 5.5 mmHg at baseline to 12.4 ± 4 mmHg at 2 years (excluding those patients who underwent surgical revision). The mean number of postoperative medications was 0.11 ± 0.4 (range, 0–3). Regarding adverse postoperative events, postoperative interventions were frequent with 43% undergoing suture manipulation, 5.1% requiring conjunctival re-suturing, 16% needing bleb needling, and 27% requiring more subconjunctival 5-fluorouracil (5-FU) postoperatively. Moreover, 31% of their patients needed cataract surgery within 2 years. In our study, only 4.5% of the patients needed further postoperative interventions, with one needing anterior chamber washout and two needing trabeculectomy.

Consistent with observations reported by other authors, our findings indicate that trabeculectomy, when compared with MIGS, achieves higher IOP reduction; however, this benefit is accompanied by higher risks and complication rates (24). Furthermore, our study demonstrated that combining KDB goniotomy with phacoemulsification results in a minimal proportion of patients requiring further intervention within 2 years.

Despite the very promising results, our study has, however, some limitations as well. The number of patients, although comparable to other studies, is not that high. Furthermore, the retrospective nature of the study and the fact that there were no predetermined guidelines on when to add or remove IOP-lowering drops can introduce some variability between patients.

## Conclusion

This study shows that eyes treated with 180-degree phacogoniotomy with KDB demonstrate significant and sustained reduction in IOP and medication burden with minimal complications. Further research with larger patient cohorts and longer postoperative follow-ups could investigate whether this procedure could sustain the target IOP and prevent the need for more invasive glaucoma procedures.

## Data Availability

The raw data supporting the conclusions of this article will be made available by the authors, without undue reservation.
